# The impact of affect labelling on responses to aversive flying-cues

**DOI:** 10.1371/journal.pone.0194519

**Published:** 2018-04-19

**Authors:** Michelle Azoum, Gavin I. Clark, Adam J. Rock

**Affiliations:** School of Behavioural, Cognitive and Social Sciences, University of New England, Armidale, NSW, Australia; Brown University, UNITED STATES

## Abstract

Individuals with flying phobia experience increases in subjective anxiety in response to flying-related cues. However, the cognitive processes that contribute to cue-reactive anxiety in individuals with flying-related anxiety remain poorly understood. Preliminary research suggests that changes in visual imagery and volitional control may contribute to this cue-reactive anxiety. Engaging in affect labelling during exposure therapy has been shown to reduce cue-reactive anxiety in individuals with fears relating to a variety of stimuli but has not been investigated in the fear of flying. The present study recruited 110 participants with a range of flying-related anxiety scores to complete an online cue-reactivity experiment. The study sought to evaluate whether an aversive flying cue triggered changes in imagery, volitional control and anxiety, and whether changes in imagery and volitional control predicted level of cue-reactive anxiety. Participants were randomly allocated to an affect labelling or non-affect labelling condition to additionally assess whether engaging in labelling one’s emotion following exposure to an aversive flying cue would attenuate cue-reactive changes in anxiety relative to a group who did not. Significant cue-reactive changes in anxiety, and volitional control were observed from neutral to aversive flying cue were observed. After accounting for the effects of flying anxiety severity, only volitional control significantly improved the prediction of cue-reactive anxiety. Participants in the affect labelling condition reported significantly smaller increases in anxiety than the non-affect labelling group following exposure to the aversive flight cue. This is the first study to indicate affect labelling may help to regulate aspects of cue-reactive anxiety in response to aversive flying stimuli.

## Introduction

Flying phobia is classified as a situational specific phobia in the Diagnostic Manual of Mental Disorders Fifth Edition (DSM-5 [1]). The criteria suggest that individuals with flying phobia experience a marked increase in fear or anxiety in anticipation of, or during, a flight-related situation [1]. A variety of studies indicate that the yearly prevalence rate of flying phobia is between 2.5% and 40%. The lower estimate may represent a clinically significant phobia whereas the higher estimates are indicative of varied levels of fear of flying [2–4]. Up to 30% of people with flying phobia may avoid flying altogether while others may restrict flying to a bare minimum or continue to endure flights with mild to significant affective and physiological distress [3–4]. Consequently, having a fear of flying can adversely impact on personal wellbeing, relationships and restrict professional opportunities where flying is required [3]. Despite the impact on personal wellbeing and high prevalence, there has been a relative lack of research pertaining to fear of flying. Indeed, Oakes and Bor [5] noted that treatments for flying phobia have been developed in the absence of the empirically driven research of causal and maintenance factors that has taken place for other disorders. Indeed, despite the fact that anxiety-related disorders display a number of common processes of maintenance [6] and are addressed through common treatment techniques [7], many of the processes which have been demonstrated to maintain anxiety responses across disorders have not been investigated in relation to flying phobia.

According to the cognitive behavioural perspective, a tendency to perceive internal and external stimuli as threatening is a core component of the maintenance of anxiety disorders [8]. Examples of external stimuli associated with anxiety responses in flying phobia include aeroplanes, turbulence, flight-related sounds and safety announcements [4]. Internal stimuli could include changes to heart rate, the vestibular system, breathing and cognition [9–10]. A number of authors have discussed flying phobia in relation to classical conditioning models of fear acquisition [11], which suggest that flying stimuli and/or a variety of interoceptive stimuli may come to elicit a fear response following initial learning experiences. This is consistent with cognitive behavioural conceptualisations of anxiety disorders which suggest perceived threat may be underpinned by a cue-reactive response, where automatically occurring cognitive processes are prompted by salient cues, and rapid increases in affective distress and behavioural responses ensue [8]. Clark and Rock [12] proposed that research should determine whether pertinent cognitive processes (e.g., mental imagery) that have been empirically tested in the context of other disorders (e.g., social phobia [13]) contribute to flying-related anxiety. Such research would help to determine whether these cognitive processes play a role in the maintenance of flying phobia in order to determine whether such processes should be targeted in treatment [12].

In addition, flying phobia treatments typically involve a combination of exposure therapy and cognitive behavioural techniques [5]. In a comprehensive review of psychological interventions for specific phobias, exposure therapies were found to be efficacious in the treatment of flying phobia [14–15]. Many studies evaluate the efficacy of treatments on the basis of whether a person took a successful flight at a follow-up 6 months to 3.5 years later [16]. However, the choice to fly does not serve as an ideal proxy of reduction in flying anxiety, as many individuals continue to take flights successfully whilst enduring significant distress [17], i.e., flying anxiety does not necessarily resolve as a result of exposure to flying scenarios. While these studies indicate exposure therapy is already an effective treatment for flying phobia, recent research suggests training people to label their emotions (i.e., *affect labelling*) during exposure to fear-relevant cues may attenuate anxiety as it occurs in phobic disorders [18–19] and in response to negatively valenced stimuli in non-clinical samples [20–21]. To date, affect labelling has not been tested in relation to cue-reactive responses to flying-related stimuli.

### Cue-reactive anxiety

Studies consistently demonstrate that samples of flying phobics and non-clinical samples experience an increase in cue-reactive anxiety in response to a range of flight-related stimuli, particularly where these depict scenarios or stimuli associated with common flying-related fears (e.g., plane crashes, planes encountering turbulence [22–24]). Despite the fact that much of this research has focused upon clinical samples of “flying phobics”, it is arguably more ecologically valid and meaningful to study flying-related anxiety on a continuum rather than within dichotomous phobic-versus-non-phobic groups. Research suggests that some degree of flying-related anxiety is reported by up to 40% of individuals [3] and studies utilizing questionnaire measures which quantify the degree of anxiety associated with the flying experience typically report a range of flying anxiety scores [24]. This suggests that many people will present with mild-to-moderate flying-related anxiety as opposed to no flying anxiety or DSM-5 diagnosable levels of flying anxiety. Consequently, it is of value to study individuals’ responses to flying-related cues within samples with a range of flying anxiety severity as it would be expected that the degree to which individuals are anxious about flying (i.e., flying anxiety severity) would predict the magnitude of cue-reactive anxiety. In support of this contention it has been demonstrated that scores on the *Flight Anxiety Situations Questionnaire* (i.e., a measure of flying anxiety severity) are positively associated with anxiety in response to aversive flying-related cues i.e., cues depicting aspects of flying typically reported as concerning by flying phobics [24]. *Cue-reactivity* is defined as a response to cues in the environment that induces increases and decreases in physiological, cognitive and affective responses [25–26]. Cue-reactivity paradigms typically involve exposing participants to a neutral cue and, subsequently, a salient cue (i.e., aversive flight-related) and measuring anxiety at each temporal-point. A cue-reactive effect is detected when there is a significant increase in, for example, anxiety from neutral to flight cue whilst controlling for baseline anxiety. In flying phobia, cue-reactivity has been demonstrated via measuring increases in self-reported distress and physiological markers of distress (e.g., heart rate and skin conductance [23, 27–28]). For instance, in a sample of flying phobics, increases in subjective anxiety (though not physiological reactivity) were reported after viewing a flight safety demonstration video as compared to when viewing a neutral video [23]. Similarly, flying phobics exposed to a flight-related video as part of a treatment trial demonstrated increases in fear as measured by subjective report and respiratory sinus arrhythmia (indicating heart rate variability) as compared to when viewing a neutral video [28].

The aforementioned studies demonstrate that anxiety is reliably cued by salient flight-related cues, with the magnitude of this increase being contingent upon participants’ flying-anxiety severity. However, Harvey, Watkins, Mansell and Shafran [29] suggested that attending to internal stimuli also cues threat-perception. There is evidence to suggest people with flying phobia attend more readily to internal sources of flight-related threat than those without a fear of flying [11]. For example, using a dichotomous listening task, Bogaerde et al. [11] found participants with higher flying anxiety recalled more internal threat-words than a control group. Bogaerde and colleagues’ study had a number of limitations, which included its small sample size (*N =* 25), a significance difference in sex ratios between experimental groups and the fact that the sample was composed of very young adults (all participants under 21 years of age) yet, nevertheless, the between-group difference effect size was large.

Research suggests that attending to physiological arousal seems to be important to the maintenance of flying phobia [30]. However, internal stimuli may adopt the form of cognitive responses including intrusive thoughts and distressing mental imagery. Little research has attempted to measure cognitive responses to flight-related cues and how such response may impact subjective distress. It has been hypothesised that changes in phenomenological variables (i.e., subjective experience) may contribute to flying phobia and associated cue-reactive anxiety [12]. Two such phenomenological variables may be visual mental imagery and volitional control.

### Mental imagery

A considerable volume of research demonstrates that vivid, distressing and spontaneous mental imagery occurs in response to fear-relevant cues and contributes to the maintenance of anxiety disorders [8, 31]. *Mental imagery* refers to the perceptual and sensory subjective experiences that occur in the absence of information derived directly from physical stimuli [31]. Mental imagery has been broadly studied in relation to a range of psychopathology [32–33] and has been shown to elicit greater affective responses than verbal processing of similar information [34]. In the experimental literature, spider anxious individuals report increases in vivid, distressing spontaneous imagery and a wider range of sensory modalities (e.g., body sensations that lasted longer) when self-generating an image of a spider but not a neutral image [35]. Additionally, research concerning social phobia indicates that imagery relating to social threat/fears occurs more frequently and contributes directly to increases in anxiety [13, 36].

To date, there are no published studies investigating the role of mental imagery in flying phobia despite the existence of treatment programs that emphasise reducing cue-reactive anxiety by targeting mental imagery (e.g., imagery desensitization [37]). Furthermore, reality testing thoughts and mental imagery was nominated as one of “10 golden rules” for designing treatments for fear of flying by 36 treatment facilities [38], indicating mental imagery is assumed to be an important treatment target. It is prudent, therefore, to determine if, and how, individuals with flying related anxiety experience mental imagery in response to fear-relevant cues. Given that people with a fear of flying report increases in anxiety in response to flight cues, it is reasonable to hypothesise that the presence of vivid, spontaneous mental imagery may contribute to cue-reactivity in flying phobia.

### Volitional control

In addition to mental imagery, another phenomenological variable that may be pertinent to the understanding of cue-reactivity in flying phobia is volitional control. The term volitional control, most commonly used with reference to behaviour, broadly denotes the extent to which an individual feels that they are able to control a particular aspect of experience [39–40]. A specific aspect of volitional control, which has been hypothesised to be pertinent to flying phobia is the extent to which a person feels that they are in control of their conscious experience which includes the experience of thoughts, mental imagery and attention [40]. People with higher levels of flying anxiety report loss of physical and cognitive control as a feared consequence of experiencing in-flight anxiety [10]. Research also suggests that flying phobics overestimate the likelihood of aversive flight-related events occurring [41] and report significantly more preoccupation with irrational and negative cognitions than non-fearful fliers [42]. Furthermore, Harvey et al. [29] suggests that the automaticity of attention towards threat cues, which has been implicated in flying phobia [11], may contribute to a sense of dyscontrol over one’s cognitive experience. In the context of flying phobia, this research may suggest decreases in perceived volitional control over cognitive processes contribute to increases in anxiety in response to flight-related cues.

To date, one study has investigated cue-reactive anxiety, mental imagery and volitional control in relation to flying-related anxiety. Utilising a cue-reactivity paradigm in a large sample presenting with a range of flying anxiety scores, Clark et al. [24] found that cue-reactive anxiety and mental imagery significantly increased, whereas volitional control significantly decreased, following exposure to aversive flight cues. Additionally, it was reported that flying anxiety severity significantly predicted cue-reactive anxiety. Interestingly, the inclusion of mental imagery and volitional control significantly improved the prediction of cue-reactive anxiety, implying that these cognitive processes are involved in the generation of cue-reactive anxiety in flying phobia. The present study extended Clark et al. [24] by investigating the impact of affect labelling on the cue-reactive response to aversive flying-related cues.

### Affect labelling

Disconfirmation of threat through exposure/behavioural experiments is regarded as a crucial component of successful treatment in flying phobia [28]. A promising strategy for optimizing exposure is *affect labelling*, which simply refers to putting one’s feelings into words in response to a fear-provoking stimuli [18].

Numerous studies have demonstrated that labelling emotions leads to decreases in self-reported anxiety, physiological arousal and reductions in amygdala (and other limbic system) activation, which is an area of the brain considered to be associated with fear conditioning and, subsequent, fear responding [18, 20, 43–45]. In spider fearful samples, individuals exhibit lower skin conductance responses when exposed to negative images of spiders with affective labels compared to images presented without labels [44]. Similarly, Kircanski et al. [18] demonstrated that spider fearful individuals trained to label their emotions took more steps toward a spider and demonstrated significant reductions in skin conductance and heat-rate. The affect labelling group in this study also maintained reductions in anxiety to a greater extent at a one week follow-up than other groups who used alternative strategies (i.e., reappraisal and distraction [18]). Similarly, affect labelling has been shown to produce greater reductions in emotional arousal in people with public speaking anxiety immediately after a public speaking task compared to a phobic group who did not engage in affect labelling [19]. No published data exists to indicate whether individuals who engage in affect labelling following exposure to an aversive flying cue would display an attenuated anxiety response relative to a group who did not.

### Present study

The present had three aims. Firstly, in order to establish whether the stimuli employed elicited a cue-reactive effect, the study aimed to evaluate whether cue-reactive changes in anxiety, mental imagery and volitional control would be demonstrated in response to an aversive flying-related cue. The second aim was to test whether the findings of Clark et al. [24] could be replicated and mental imagery and volitional control would improve the prediction of cue-reactivity anxiety after accounting for the effects of flying anxiety scores. The final aim was to compare two groups to investigate whether labelling one’s emotions in response to an aversive flight-related cue was associated with lower self-reported anxiety. The following predictions were made:

*H1*: Anxiety would increase from neutral cue to flight cue in the overall sample, controlling for baseline anxiety.
*H2*: Mental imagery would increase from neutral cue to flight cue in the overall sample, controlling for baseline imagery.
*H3*: Volitional control would decrease from neutral cue to flight cue in the overall sample, controlling for baseline measures on these variables.
*H4*: Cue-reactive mental imagery and cue-reactive volitional control would improve the prediction of cue-reactive anxiety in the overall sample after accounting for the effects of flying anxiety severity.
*H5*: There would be a greater increase in cue-reactive anxiety from neutral cue to flight cue in the non-affect labelling group, controlling for flying anxiety severity.

## Method

### Participants

An a priori power analysis determined 77 participants were required to detect a medium effect size (*f*^*2*^ = .15), with three predictors at a .8 power level. Inclusion criteria stipulated that participants were over the age of 18, proficient in reading English and resided within Australia. A total of 280 people commenced the online experiment. Twenty-eight participants were excluded for not meeting the inclusion criterion of residing in Australia and 102 dropped out prior to completion. We note that this level of attrition is equivalent to other online experimental studies requiring similar participation time [46–47].

Forty people were excluded from the final sample due to non-engagement with the tasks, measured by time spent on the neutral and/or flight scenario pages of less than 50 seconds (the minimum time estimated to be required to complete the task), with some spending as little as 2 seconds. The final sample consisted of 110 people with a mean age of 39.8 years (*SD* = 13.15) with 17 males with a mean age of 43.65 (*SD* = 15.88) and 93 females with a mean age of 39.1 (*SD* = 12.56).

### Materials and measures

The following materials and measures were presented within an online experiment utilizing Qualtrics^TM^ as a host site [48].

#### Demographic questions

Participants were asked to report their sex, age, martial status, education level, number of flights per year and the length of time since their last flight. Demographic information is presented in Table 1.

**Table 1:** Demographic information (N = 110).

Variable	Frequency
Marital Status	
Single/never married	22
Married/domestic partnership	77
Widowed	2
Divorced	5
Separated	4
Education	
Schooling to Grade 10	9
Schooling to Grade 12	15
Diploma (e.g. trade)	19
Undergraduate Degree	43
Postgraduate Degree	24
Average no. of flights taken per year	
0	11
1–2	69
3–5	19
6–10	10
10+	1
Last flight on an aeroplane	
In last week	7
In the last month	14
1–3 months	25
3–6 months	8
6–12 months	32
Over 12 months ago	24

#### Flight anxiety situations questionnaire

(FAS [49]): The FAS consists of 32 items which yield a total anxiety score and scores on three subscales: an anticipatory flight anxiety scale (14 items), an in-flight anxiety scale (11 items) and a generalised anxiety scale (7 items). Item responses are recorded on a five point likert scale ranging from 0 (no anxiety) to 5 (overwhelming anxiety). The FAS has good internal consistency with Cronbach’s alphas ranging from .85 to .96 [50]. Cronbach’s alphas ranged from .89 to .98 in the present study.

#### Visual analogue scale–cue-reactive anxiety

(VAS-A). To measure subjective anxiety in response to cues, participants rated their level of current anxiety on a scale of 0 (no anxiety) to 100 (extreme anxiety). Visual analogue scales have been widely used in emotional regulation and cue-reactivity studies and are considered a sensitive measure [18, 51].

#### Phenomenology of consciousness inventory

(PCI [40]). Items from the PCI were utilised to retrospectively assess mental imagery and volitional control during exposure to two cues (described below). The PCI is a 53-item self-report retrospective assessment instrument allowing quantification of subjective experience in response to a stimulus. The full PCI measures 12 pertinent dimensions of phenomenology (e.g., attention, rationality). Participants are presented with paired statements on either end of a seven point dipole scale, with each pair of statements designed to detect the intensity of a phenomenological response from 0 (little or no intensity) to 6 (more present and/or very intense). The mental imagery dimension of the PCI contains four paired statements measuring the amount and vividness of mental imagery. An example of the paired statements which assess this dimension includes; “I experienced a great deal of visual imagery” versus “I experienced no visual imagery at all”. The volitional control dimension consists of three paired statements measuring perception of control over thoughts and images with an example item being “The thoughts and images I had were under my control; I decided what I thought or imagined” versus ‘‘Images and thoughts popped into my mind without my control”. The PCI has good internal consistency with Cronbach’s alphas ranging from .70 to .90. In addition, the PCI has good criterion validity demonstrated by reliability discriminating between different phenomenological experiences [52]. Cronbach’s alphas in the present study ranged from .70 to .91.

#### Neutral and flight-related scenarios

The present study utilised the imagery induction vignettes employed by Clark et al. [24]. Vignettes have been employed in a number of studies with the aim of generating salient first person perspectives of imagined situations in order to evaluate changes in cognitive-affective variables [53–54]. The flying-related vignette, henceforth labelled the “flight cue” was designed to elicit imagery of experiences that have been reported to be anxiety provoking for flying phobics (e.g., boarding a flight, taxi-out, take off, severe turbulence [28]). The neutral cue was designed to be matched for length and descriptive content and asked participants to imagine going to the cinema to see a film and the typical sensory input expected. Previous research has demonstrated that this cue is not associated with a significant increase in anxiety relative to baseline measures [24]. Participants were asked to slowly read and imagine themselves in the scenarios described. Previous research has demonstrated that this flight cue elicits significant increases in anxiety, mental imagery and volitional control relative to the neutral cue [24].

We note that the presentation time for the imagery induction vignettes were not standardised due to the fact that participant reading time and ease of generating mental imagery was likely to vary as a function of individual difference variables across participants [55]. In order to ensure that the duration of time spent engaging with aversive flying imagery cues was not influenced by flying anxiety severity or the anxiety experienced in response to such cues (i.e. suggesting disengagement and avoidance), Pearson correlation coefficients were calculated between flying imagery completion time, FAS score and increase in anxiety between neutral and flying cue. We found no significant association between cue completion time and flying anxiety severity, *r* = 0.07, *n* = 110, *p* = 0.503. Similarly we found no significant association between cue completion time and anxiety in response to the flying cue *r* = 0.08, *n* = 110, *p* = 0.424.

#### Affect labelling

Instructions for participants to engage in affect labelling were adapted from Kircanski et al. [18]. Participants in the affect labelling group were instructed to write a sentence about their emotional experience while imagining the flight scenario. To ensure task adherence the sentences participants typed were included as part of data collection.

### Design

This study employed a cue-reactive paradigm using a 2 x 3 mixed design with the between-subjects factor, group, consisting of two levels (affect labelling and non-affect labelling) and the within-subject factor, cue, consisting of three levels (baseline, neutral and flight). In accordance with previously established cue-reactivity protocols, the cues were not counterbalanced in order to ensure that participant response to the salient cue would not carry over to the neutral cue [26].

### Procedure

Ethics approval for the present study was obtained from the University of New England’s Human Research Ethics Committee. An invitation to participate was posted on social media forums with a brief description and a link to the experiment hosted on Qualtrics. Students enrolled in introductory psychology courses from the University of New England were given credit points for participating. Prospective participants who clicked on the link were presented with a study information sheet and asked if they consented to participate. Participants completed the demographics questionnaire, FAS and answered the baseline VAS-A and PCI items based on their last five minutes of experience. Subsequently, participants completed the VAS-A and PCI items in response to the neutral cue. At a further subsequent time, participants were randomly assigned to the affect labelling (AL) or non-affect labelling (non-AL) groups. All participants were presented with the flight cue. Participants in the affect labelling group were instructed to type a sentence regarding their emotional response to the flight cue prior to answering VAS-A and PCI items. The non-AL group simply completed the VAS-A and PCI items in response to the flight cue.

## Results

A wide range of flying anxiety severity was indicated in the current sample with FAS scores ranging from 32 to 146, indicating the recruitment of individuals with low and high flying-related anxiety. Nousi et al. [50] reported norms for the FAS in which a non-flying phobic sample’s mean score was 39.84 (*SD =* 11.92) and a sample of flying phobic patients recorded a mean score of 102.42 (*SD* = 22.48). The mean FAS score for the entire sample in the present study, 58.50 (*SD* = 28.16), therefore reflects a flying anxiety severity mean score which falls between previously established FAS norms for flying phobic and non-flying phobic samples [50]. Notably, 44% of the present sample recorded FAS scores at least one standard deviation above the mean flying anxiety norms for non-flying phobics reported by Nousi et al. [50] and 20% of the sample displayed scores consistent with the FAS norms reported for the flying phobic patients sample. Thus the present sample can be claimed to reflect a continuum of flying anxiety severity. A series of independent *t-*tests indicated that there were no significant differences between FAS score in the AL (*M =* 56.79, *SD =* 28.10) and non-AL group (*M =* 60.32, *SD =* 28.38) or any differences between groups on any demographic measures, suggesting the groups were equivalent.

### Hypothesis 1

A one-way repeated measures analysis of covariance (ANCOVA) was performed to evaluate change in anxiety from neutral to flight cue, with baseline anxiety entered as a covariate. There was a statistically significant main effect of cue, *F*(1,108) = 21.074, *p* < .001, partial η^2^ = .163, thus, *H1* was supported.

### Hypothesis 2

In order to determine whether the imagery induction cues successfully led to the generation of mental imagery two paired sample t-tests were carried out to measure change between imagery at baseline and imagery at neutral and flying cue. Participants reported significantly greater amounts of mental imagery at neutral cue (*M* = 3.96, *SD* = 1.47) relative to baseline (*M* = 3.54, *SD* = 1.53), *t*(109) = 3.78, *p* < .001. Participants also reported significantly greater amounts of mental imagery at flying cue (*M* = 4.13, *SD* = 1.38) relative to baseline (*M* = 3.54, *SD* = 1.53), *t*(109) = 5.37, *p* < .001, thus indicating the cue-induction was successful. A one-way repeated measures ANCOVA was performed to evaluate change in imagery from neutral to flight cue, with baseline imagery entered as a covariate. After controlling for baseline there was no main effect of cue for the overall sample, *F*(1,108) = .230, *p* = .633, partial η^2^ = .002. *H2* was not supported.

### Hypothesis 3

A one-way repeated measures ANCOVA was performed to evaluate change in volitional control from neutral to flight cue, with baseline volitional control entered as a covariate. After controlling for baseline there was a significant main effect for cue, *F*(1,108) = 6.161, *p* = .015, partial η^2^ = .054, thus, *H3* was supported. Descriptive statistics for *H1*, *H2* and *H3* are presented in Table 2.

**Table 2:** Means and standard deviations for PCI and VAS anxiety for neutral-cue, flight-cue and difference scores by group and overall sample.

	Neutral-cue		Flight-cue		Difference Scores	
	Mean	SD	Mean	SD	Mean	SD
Affect Labelling (*n* = 57)						
Anxiety	7.4	10.68	25.29	26.46	17.87	28.14
Mental Imagery	4	1.46	4.28	1.34	.28	1.19
Volitional Control	5	1.25	4.44	1.35	-.56	1.48
Non-Affect Labelling (*n* = 53)						
Anxiety	6.53	12.2	34.94	27.62	28.42	26.81
Mental Imagery	3.92	1.49	3.96	1.42	.04	1.27
Volitional Control	4.5	1.41	3.9	1.5	-.60	1.28
Sample (*N* = 110)						
Anxiety	6.99	11.39	29.94	27.34	22.96	26.87
Mental Imagery	3.96	1.47	4.13	1.39	.17	1.22
Volitional Control	4.75	1.35	4.18	1.44	-.58	1.38

*Note*. Difference scores were calculated by subtracting neutral-cue scores from flight-cue scores.

### Hypothesis 4

A hierarchical multiple regression was conducted to test *H4*. A difference score was calculated for each variable by subtracting scores on the neutral cue from the flight cue. Means and *SD*s for the difference scores are presented in Table 2. At Step 1, FAS scores significantly predicted anxiety difference scores (i.e., cue-reactive anxiety) accounting for 30.8% of the variability, *R*^*2*^ = 0.308 (Adjusted *R*^*2*^ = 0.301), *F*(1,108) = 48.042, *p* < .001. The inclusion of mental imagery and volitional control difference scores at step 2 significantly improved the prediction of anxiety scores by 5.1%, Δ*R*^2^ = 0.051, *R*^2^ = .358 (Adjusted *R*^*2*^ = 0.340), *F*(3, 106) = 19.740, *p* = .018. At both steps of the model the *R* value was significantly different from zero (step 1, *R* = .555, *F*(1,108) = 48.042, *p* < .001 and step 2, *R* = .599, *F*(3, 106) = 19.740, *p* < .001). Altogether the variance explained by the model was 35.8%. An examination of the beta coefficients (presented in Table 3) revealed that volitional control was the only significant predictor of cue-reactive anxiety in the final regression model (*sr*^*2*^ = .048) after accounting for FAS scores, thus, *H4* was partially supported.

**Table 3:** Unstandardised (B) and standardised (β) regression coefficients and squared semi-partial correlations (sr^2^) for each predictor variable at each step of the hierarchical multiple regression predicting anxiety difference scores.

Variable	*B* [95% CI]	β	*sr*^*2*^
Step 1			
FAS	.529 [.378, .681]**	.555	.308
Step 2			
FAS	.466 [.309, .623]**	.489	.209
Mental Imagery	-.761 [-4.196, 2.675]	-.035	-.001
Volitional Control	-4.489 [-7.646, -1.332]*	-.231	-.048

*Note*. CI = confidence interval

** *p* < .001

* *p* < .05.

### Hypotheses 5 and 6

**Fig 1:**
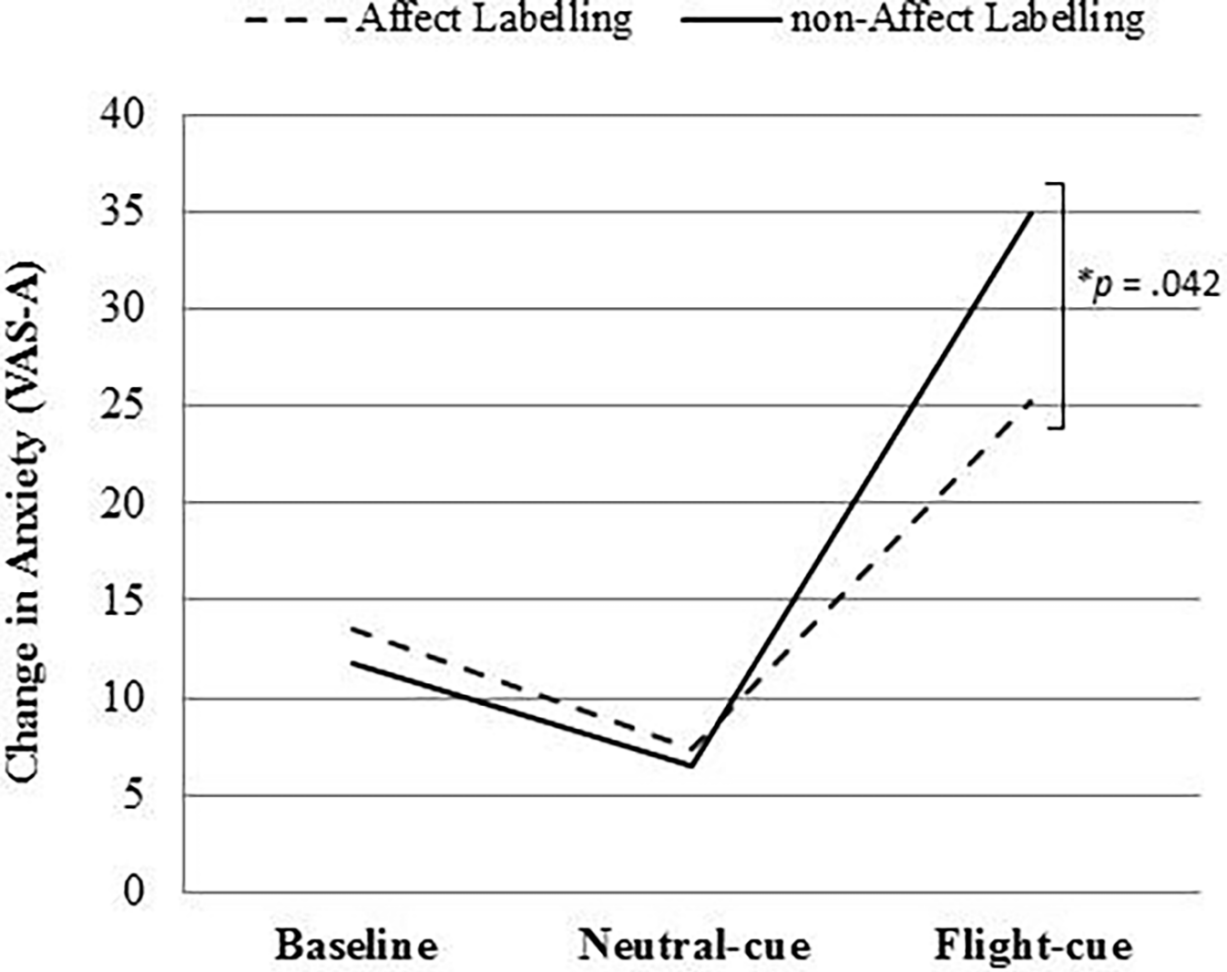
Change in anxiety scores by group at baseline, immediately following the neutral cue and following the flight cue. *Differences were significant following flight cue after accounting for the effects of flight anxiety severity.

*H5* was evaluated by a one-way between groups ANCOVA with FAS scores entered as the covariate. There was a statistically significant difference between the AL and non-AL group on cue-reactive anxiety, *F*(1,107) = 4.231, *p* = .042, partial η^*2*^ = .038. The adjusted group means revealed people in the non-AL group (*M* = 27.46, *SE* = 3.04) reported greater mean increases in anxiety in response to the flight cue than the AL group (*M* = 18.76, *SE* = 2.93). Thus, *H5* was supported. Fig 1 provides a graphical representation of the results for anxiety differences from baseline to flight cue.

As noted above, the nature of the cue-reactive methodology dictates that the phenomenological variables of interest (imagery and volitional control) were evaluated based on participant retrospective report regarding their experience during cue-exposure [51]. As such, it would not be expected that the affect labelling task, which took place immediately following the cue exposure, would impact upon these variables. Consistent with this conceptualisation, no differences were found between groups on cue-reactive volitional control, *F* (1,107) = .005, *p* = .944 or mental imagery, *F* (1,107) = 1.333, *p* = .251.

## Discussion

The first aim of this study was to evaluate whether the stimuli employed elicited a significant cue-reactive effect. In accordance with *H1* a significant increase in anxiety was observed from neutral cue to flight cue in the overall sample. Contrary to previous research findings investigating cue-response in specific phobia [35], there was no significant difference for mental imagery scores from neutral cue to flight cue (*H2*). However, we note that the difference was in the hypothesised direction. The imagery induction scenarios utilised in this study have reliably demonstrated an increase in anxiety from neutral to flight cue in previous research [24]. However, because the neutral scenario involves induction of mental imagery, the PCI items may not be sensitive enough to capture increases in spontaneously occurring mental imagery when participants are already generating mental imagery. The sample was also non-clinical. Consequently, it may be that the occurrence of mental imagery differs in clinically significant flying phobia. It is important for future research to address these measurement concerns to determine the role of imagery, as it is already considered an important target in flying phobia treatment [38]. Consistent with *H3* a significant cue-reactive decrease in volitional control was observed. This is consistent with the proposal that exposure to an aversive flying cue will result in changes in the extent to which individuals feel in control of their conscious experience [24].

Whilst the nature of an online experimental design creates some uncertainty regarding the extent to which individuals engage with the study materials, the results of the study confirm that the experimental manipulation was successful. Firstly, the sample demonstrated significant increases in anxiety from neutral to flying cue, indicating that the flight cue successfully provoked anxiety (and by inference, perception of threat) across participants. Both neutral and flight imagery cues resulted in significant increases in self-reported mental imagery across participants relative to baseline, confirming that each resulted in the generation of mental imagery. Whilst the results did not demonstrate the hypothesized increase in imagery from neutral to flight cue, the decrease in volitional control from neutral to flying cue supports the hypothesized perceived loss of control regarding the content of conscious experience between neutral and flying cue. Collectively, these results confirm the success of the within-group experimental manipulation.

In accordance with *H4*, flying anxiety severity was found to positively predict cue-reactive anxiety. This result is consistent with the findings of previous studies in flying phobia that have found that greater increases in cue-reactive anxiety are positively associated with flying anxiety severity [24]. The inclusion of mental imagery and volitional control significantly improved the prediction of cue-reactive anxiety in response to flight-related cues. An important finding was that decreases in the subjective sense of volitional control significantly improved the prediction of cue-reactive anxiety by 4.8% after accounting for the effects of flying anxiety severity. Previous research has found that individuals with flying phobia self-report fearing the loss of cognitive control [10]. The current study suggests that this perceived loss of control can be elicited in response to aversive flying cues and that decreases in a sense of control of thoughts and imagery may be contribute to increases in anxiety in response to flying cues. Although no causal inferences can be drawn from the present study, these findings are consistent with the hypothesis that automatically occurring changes in conscious experience in response to fear-cues might be contributing to perception of threat in flying phobia [12, 29]. Consequently, if people experience a decrease in their sense of control over their conscious experience in response to fight-cues, they may interpret their subjective experience as evidence that a threat is present, which may contribute to maintenance of the disorder [12].

The final aim of this study was to investigate whether labelling one’s emotions in response to an aversive flight-related cue was associated with lower self-reported anxiety and whether affective labelling impacts on the subjective experience of mental imagery and volitional control. Participants in the AL group reported significantly smaller increases in anxiety than the non-AL group. This finding is consistent with previous experiments where lower subjective anxiety was reported in response to negatively valenced stimuli when participants engaged in affect labelling [20, 45]. This is the first study indicating that affect labelling may be a viable technique to reduce cue-reactive anxiety in flying phobia (discussed below).

As the first study to evaluate the impact of affect labelling on cue-reactive response to an aversive flying related cue the results are of significant importance. Nevertheless it should be noted, that the design of the study was such that the relative effect of an affect labelling versus non-affect labelling condition could only be evaluated through a comparison between groups at a single time point. In contrast, to date the impact of affect labelling has typically been measured through the attenuation of anxiety over time [44]. For example, in previous research affect labelling was shown to have potentially lasting effects on behaviours (e.g., approaching a feared stimuli) and physiological markers of distress (e.g., skin conductance) for spider fearful individuals during follow-up tests [18, 44]. Future research should establish if the impact of affect labelling can be demonstrated with individuals with a diagnosis of flying phobia and whether it can be demonstrated to have a lasting effect on flying-related fear responses. Such research should aim to establish whether attenuation occurs over multiple trials and time points and in relation to actual flying stimuli.

Previous research also indicates that affect labelling reliably contributes to reductions in physiological arousal during exposure to feared stimuli [19, 44]. As attending to physical arousal symptoms of anxiety in flying phobia may be an important contributor to fear responses [30], it would be beneficial for future research to test whether attenuation of physiological arousal mediates the relationship between labelling emotions and decreases in anxiety in flying phobia. As noted above, the nature of the methodology does not allow us to make any conclusions regarding whether affect labelling would impact upon the experience of the phenomenological variables of interest during cue exposure. Future research should therefore aim to modify the cue-reactivity paradigm in order to determine whether engaging in affect labelling during cue-exposure impacts upon changes in volitional control and mental imagery.

Lastly, experiments comparing affect labelling and reappraisal (i.e., reinterpreting the meaning of a situation to alter an emotional response) techniques consistently demonstrate the neural response patterns and self-reported anxiety scores of participants are remarkably similar when responding to aversive stimuli [20, 43, 45]. This indicates the tasks may be targeting similar cognitive processes. Despite psychological interventions in flying phobia including cognitive reappraisal [5], there is no reference to any utilising affect labelling. It would be important for future research to compare the effectiveness of these two strategies for flying phobia as has been done in the wider affect labelling literature to determine whether affect labelling could results in improved outcomes relative to exposure which does not incorporate affect labelling.

### Limitations

There are a number of limitations to this study in addition to those highlighted above. A convenience sample from the general population was utilised and, therefore, it is uncertain to what degree these results apply to people meeting diagnostic criteria for flying phobia in the DSM-5 [1]. Notably, the sample did show significant increases in cue-reactive anxiety, which is consistent with studies indicating that some level of flying anxiety is common amongst the general population [3]. Participants also needed to opt-into the study and then choose to participate. The sample exhibited a high drop-out rate, thus, the possibility of selection bias needs to be considered. There is a possibility that those who were less likely to opt-in or complete the study differed in some way from those who did (e.g., people with clinically significant flying phobia may be less likely to participate). It is also important to note that the study sample was comprised of 84% female participants. Equivalent gender skews have been observed in a variety of online psychology studies conducted in Australia, which have similarly involved the recruitment of self-selecting volunteer participants [56–57]. The fact that similar gender ratios have been observed in a variety of studies may infer that this over-representation of female participants may not be due to the study topic or the variables assessed. Nevertheless, such differences in male-to-female representation limits the extent to which the results may be seen as generalisable to those who identify as male.

Whilst online experimental studies have been utilised extensively within the literature [46–47] this medium presents an inherent limitation in that there is a lack of control over testing and participant engagement with test stimuli. Future extensions of this study should therefore aim to examine these variables within more strictly controlled laboratory conditions. Finally, the cues used in the present study were an imagery induction exercise and not representative of all cues individuals would encounter in the real world. Improvements could be made to the ecological validity of the cues by introducing more sensory information. For example, sound has been found to elicit higher anxiety reporting in experiments carried out in laboratory settings [27] and disruption to the vestibular system caused by plane movement is reported as highly anxiety provoking in flying phobics [3]. Nevertheless, significant results were detected in the present sample demonstrating the strength of the associations between cues and the variables measured. A final consideration in relation to ecological validity is that the flying imagery scenario was somewhat aversive in that it described a plane encountering significant turbulence and adverse weather and, consequently, may not reflect the cue-response to general flying cues (e.g. a non-eventful take-off).

### Conclusion

The present study supports previous findings suggesting that level of flying anxiety severity predicts increases in anxiety in response to aversive flight cues. In addition, the study provides further experimental data indicating that the subjective sense of loss of volitional control may contribute to the level of cue-reactive anxiety experienced. Finally, this study is the first to provide preliminary evidence that the aforementioned cue-reactive anxiety response may be attenuated by labelling emotions. Affect labelling may be a promising technique to utilise in the treatment of flying phobia and warrants further research to investigate efficacy of this method over time, physiological responses and with a clinical population.

## Supporting information

S1 File Dataset. (SAV)

## References

[1] American Psychiatric Association. Diagnostic and statistical manual of mental disorders- Fifth Edition (DSM-5). Arlington, VA: American Psychiatric Publishing; 2013.

[2] Ekeberg Ø, Seeberg I, Ellertsen BB. The prevalence of flight anxiety in Norway. Nordic Journal of Psychiatry. 1989; 43(5):443–448. doi: 10.3109/08039488909107869

[3] Oakes M, Bor R. The psychology of fear of flying (part I): A critical evaluation of current perspectives on the nature, prevalence and etiology of fear of flying. Travel Medicine and Infectious Disease. 2010a; 8(6):327–338. 21050826 10.1016/j.tmaid.2010.10.001

[4] Van Gerwen LJ, Spinhoven P, Diekstra RF, Van Dyck R. People who seek help for fear of flying: Typology of flying phobics. Behavior Therapy. 1997; 28(2):237–251. doi: 10.1016/S0005-7894(97)80045-7

[5] Oakes M, Bor R. The psychology of fear of flying (part II): A critical evaluation of current perspectives on approaches to treatment. Travel Medicine and Infectious Disease. 2010b; 8(6):339–363. doi: 10.1016/j.tmaid.2010.10.002 21071281 10.1016/j.tmaid.2010.10.002

[6] McManus F, Clark G, Shafran R, Muse K. A preliminary evaluation of a transdiagnostic approach to treating co-morbid anxiety disorders. Behavioural and Cognitive Psychotherapy. 2015; 43:744–758. doi: 10.1017/S1352465814000435 25362937 10.1017/S1352465814000435

[7] Clark GI, Hanstock TL, Clark LH. Psychological treatment of co-occurring anxiety disorders in clinical practice: A vignette study. Australian Psychologist. 2016 doi: 10.1111/ap.12214

[8] Clark DM. Anxiety disorders: Why they persist and how to treat them. Behaviour Research and Therapy. 1999; 37:5–27. doi: 10.1016/S0005-7967(99)00048-0 10.1016/s0005-7967(99)00048-0 10402694

[9] Van Almen KLM, Van Gerwen LJ. Prevalence and behavioral styles of fear of flying. Aviation Psychology and Applied Human Factors. 2013; 3: 39–43. doi: 10.1027/21920923/a000035

[10] Wilhelm FH, Roth WT. Clinical characteristics of flight phobia. Journal of Anxiety Disorders. 1997; 11(3):241–261. doi: 10.1016/S0887-6185(97)00009-1 9220299 10.1016/s0887-6185(97)00009-1

[11] Bogaerde AV, Pieters J, De Raedt R. The nature of threat: Enhanced recall of internal threat words in fear of flying. Cognitive Therapy and Research. 2012; 36:390–396. doi: 10.1007/s10608-010-9346-7

[12] Clark GI, Rock AJ. Processes contributing to the maintenance of flying phobia: A narrative review. Frontiers in Psychology. 2016; 7(754):1–21. doi: 10.3389/fpsyg.2016.00754 27313550 10.3389/fpsyg.2016.00754 PMC4887486

[13] Hirsch C, Meynen T, Clark D. Negative self-imagery in social anxiety contaminates social interactions. Memory. 2004; 12:496–506. doi: 10.1080/09658210444000106 15487545 10.1080/09658210444000106

[14] Choy Y, Fyer AJ, Lipsitz JD. Treatment of specific phobia in adults. Clinical Psychology Review. 2007; 27(3):266–286. doi: 10.1016/j.cpr.2006.10.002 17112646 10.1016/j.cpr.2006.10.002

[15] Wolitzky-Taylor KB, Horowitz JD, Powers MB, Telch MJ. Psychological approaches in the treatment of specific phobias: A meta-analysis. Clinical Psychology Review. 2008; 28(6):1021–1037. doi: 10.1016/j.cpr.2008.02.007 18410984 10.1016/j.cpr.2008.02.007

[16] Van Gerwen LJ, Spinhoven P, Diekstra RF, Van Dyck R. Multicomponent standardized treatment programs for fear of flying: Description and effectiveness. Cognitive and Behavioral Practice. 2002; 9(2):138–149. doi: 10.1016/S1077-7229(02)80007-4

[17] Öst LG, Brandberg M, Alm T. One versus five sessions of exposure in the treatment of flying phobia. Behaviour Research and Therapy. 1997; 35(11):987–996. doi: 10.1016/S0005-7967(97)00077-6 9431728 10.1016/s0005-7967(97)00077-6

[18] Kircanski K, Lieberman MD, Craske MG. Feelings into words contributions of language to exposure therapy. Psychological Science. 2012; 23(10):1086–1091. doi: 10.1177/0956797612443830 22902568 10.1177/0956797612443830 PMC4721564

[19] Niles AN, Craske MG, Lieberman M., Hur C. Affect labeling enhances exposure effectiveness for public speaking anxiety. Behaviour Research and Therapy. 2015; 68:27–36. doi: 10.1016/j.brat.2015.03.004 25795524 10.1016/j.brat.2015.03.004

[20] Burklund LJ, Creswell JD, Irwin M, Lieberman M. The common and distinct neural bases of affect labeling and reappraisal in healthy adults. Frontiers in Psychology. 2014; 5(221):1–10. doi: 10.3389/fpsyg.2014.00221 24715880 10.3389/fpsyg.2014.00221 PMC3970015

[21] Lieberman MD, Eisenberger NI, Crockett MJ, Tom SM, Pfeifer JH, Way BM. Putting feelings into words affect labeling disrupts amygdala activity in response to affective stimuli. Psychological Science. 2007; 18(5):421–428. doi: 10.1111/j.1467-9280.2007.01916.x 17576282 10.1111/j.1467-9280.2007.01916.x

[22] Busscher B, Spinhoven P, Van Gerwen LJ, De Geus EJ. Anxiety sensitivity moderates the relationship of changes in physiological arousal with flight anxiety during in vivo exposure therapy. *Behaviour Research and Therapy*. 2013; 51(2):98–105. doi: 10.1016/j.brat.2012.10.009 23262117 10.1016/j.brat.2012.10.009

[23] Busscher B, Van Gerwen LJ, Spinhoven P, De Geus EJ. Physiological reactivity to phobic stimuli in people with fear of flying. Journal of Psychosomatic Research. 2010; 69(3):309–317. doi: 10.1016/j.jpsychores.2009.12.005 20708454 10.1016/j.jpsychores.2009.12.005

[24] Clark GI, Rock AJ, Hales S, Hall A. Cue-reactive imagery, volitional control and anxiety in response to aversive flying-related scenarios. 2017. Manuscript submitted for publication.

[25] Rock AJ, Kambouropoulos N. Toward a phenomenology of urge to drink: A future prospect for the cue-reactivity paradigm. North American Journal of Psychology. 2007; 9(2):387–406.

[26] Rock AJ, Kambouropoulos N. Conceptualizing craving: Extrapolations from consciousness studies. *North American Journal of Psychology*. 2008; 10(1):127–146. Retrieved from www.researchgate.net/publication/278244869

[27] Bornas X, Llabrés J, Noguera M, López AM, Barceló F, Tortella-Feliu M, Fullana MÀ. (2004). Self-implication and heart rate variability during simulated exposure to flight-related stimuli. Anxiety, Stress & Coping. 2004; 17(4):331–339. doi: 10.1080/10615800512331328777

[28] Busscher B, Spinhoven P, De Geus EJ. Psychological distress and physiological reactivity during in vivo exposure in people with aviophobia. Psychosomatic Medicine. 2015; 77(7):762–774. doi: 10.1097/PSY.0000000000000209 26186430 10.1097/PSY.0000000000000209

[29] Harvey AG, Watkins E, Mansell W, Shafran R. Cognitive behavioural processes across psychological disorders: A transdiagnostic approach to research and treatment. Oxford, UK: Oxford University Press; 2004 doi: 10.1093/med:psych/9780198528883.001.0001

[30] Bogaerde A, De Raedt R. Internal sensations as a source of fear: Exploring a link between hypoxia and flight phobia. Anxiety, Stress and Coping. 2013; 26:343–354. doi: 10.1080/10615806.2012.673592 22574671 10.1080/10615806.2012.673592

[31] Hirsch CR, Holmes EA. Mental imagery in anxiety disorders. Psychiatry. 2007; 6(4):161–165. doi: 10.1016/j.mppsy.2007.01.005

[32] Day S, Holmes E, Hackmann A. Occurrence of imagery and its link with early memories in agoraphobia. Memory. 2004; 12(4):416–427. doi: 10.1080/09658210444000034 15487538 10.1080/09658210444000034

[33] Speckens AE, Hackmann A, Ehlers A, Cuthbert B. Imagery special issue: Intrusive images and memories of earlier adverse events in patients with obsessive compulsive disorder. Journal of Behavior Therapy and Experimental Psychiatry. 2007; 38(4):411–422. doi: 10.1016/j.jbtep.2007.09.004 18005933 10.1016/j.jbtep.2007.09.004

[34] Holmes EA, Mathews A, Mackintosh B, Dalgleish T. The causal effect of mental imagery on emotion assessed using picture-word cues. Emotion. 2008; 8(3):395–409. doi: 10.1037/1528-3542.8.3.395 18540755 10.1037/1528-3542.8.3.395

[35] Pratt D, Cooper MJ, Hackmann A. Imagery and its characteristics in people who are anxious about spiders. Behavioural and Cognitive Psychotherapy. 2004; 32(2):165–176. doi: 10.1017/S1352465804001158

[36] Hirsch C.R, Mathews A, Clark DM, Williams R, Morrison JA. The causal role of negative imagery in social anxiety: A test in confident public speakers. Journal of Behavior Therapy and Experimental Psychiatry. 2006; 37(2):159–170. doi: 10.1016/j.jbtep.2005.03.003 15913541 10.1016/j.jbtep.2005.03.003

[37] Wiederhold BK, Gevirtz RN, Spira JL. Virtual reality exposure therapy vs. imagery desensitization therapy in the treatment of flying phobia In: Riva G, Galimberti C, editors. Towards cyberpsychology: Mind, cognitions and society in the internet age. Amsterdam: IOS Press; 2001 pp. 252–272. Retrieved from www.researchgate.net/publication/237246819_14

[38] Van Gerwen LJ, Diekstra RF, Arondeus JM, Wolfger R. Fear of flying treatment programs for passengers: An international update. Travel Medicine and Infectious Disease. 2004; 2(1):27–35. doi: 10.1016/j.tmaid.2004.01.002 17291954 10.1016/j.tmaid.2004.01.002

[39] Clark GI, Rock AJ, McKeith C, Coventry WJ. Cue-reactive rationality, visual imagery and volitional control predict cue-reactive urge to gamble in poker-machine gamblers. Journal of Gambling Studies. 2016 doi: 10.1007/s10899-016-9650-6 27804002 10.1007/s10899-016-9650-6

[40] Pekala RJ. Quantifying consciousness: An empirical approach. New York: Plenum; 1991.

[41] Mavromoustakos E, Clark GI, Rock AJ. Evaluating perceived probability of threat-relevant outcomes and temporal orientation in flying phobia. Plos One. 2016; 11(8):e0161272 doi: 10.1371/journal.pone.0161272 27557054 10.1371/journal.pone.0161272 PMC4996458

[42] Möller AT, Nortje C, Helders SB. Irrational cognitions and the fear of flying. Journal of Rational-Emotive and Cognitive-Behavior Therapy. 1998; 16(2):135–148. doi: 10.1023/A:1024938411949

[43] Payer DE, Baicy K, Lieberman MD, London ED. Overlapping neural substrates between intentional and incidental down-regulation of negative emotions. Emotion. 2012; 12(2):229–235. doi: 10.1037/a0027421 22468617 10.1037/a0027421 PMC4111128

[44] Tabibnia G, Lieberman MD, Craske MG. The lasting effect of words on feelings: Words may facilitate exposure effects to threatening images. Emotion. 2008; 8(3):307–317. doi: 10.1037/1528-3542.8.3.307 18540747 10.1037/1528-3542.8.3.307 PMC4727455

[45] Lieberman MD, Inagaki TK, Tabibnia G, Crockett MJ. Subjective responses to emotional stimuli during labeling, reappraisal, and distraction. Emotion. 2011; 11(3): 468–480. doi: 10.1037/a0023503 21534661 10.1037/a0023503 PMC3444304

[46] Dandurand F, Shultz TR, Onishi KH. Comparing online and lab methods in a problem-solving experiment. Behavior Research Methods. 2008; 40(2):428–434. 18522052 10.3758/brm.40.2.428

[47] McKeith CF, Rock AJ, Clark GI. Trait mindfulness, problem-gambling severity, altered state of awareness and urge to gamble in poker-machine gamblers. Journal of Gambling Studies. 2017; 33(2):617–632. doi: 10.1007/s10899-016-9635-5 27619216 10.1007/s10899-016-9635-5

[48] Qualtrics (Version 042016) [Computer software]. Provo, UT: Qualtrics. Retrieved from http://www.qualtrics.com

[49] Van Gerwen LJ, Spinhoven P, Van Dyck R, Diekstra RF. Construction and psychometric characteristics of two self-report questionnaires for the assessment of fear of flying. Psychological Assessment. 1999; 11(2):146–158. doi: 10.1037/1040-3590.11.2.146

[50] Nousi A, Van Gerwen L, Spinhoven P. The flight anxiety situations questionnaire and the flight anxiety modality questionnaire: Norms for people with fear of flying. Travel Medicine and Infectious Disease. 2008; 6(5):305–310. doi: 10.1016/j.tmaid.2008.06.001 18760254 10.1016/j.tmaid.2008.06.001

[51] Tricker C, Rock AJ, Clark GI. Cue-reactive altered state of consciousness mediates the relationship between problem-gambling severity and cue-reactive urge in poker-machine gamblers. Journal of Gambling Studies. 2016; 32(2):661–674. doi: 10.1007/s10899-015-9549-7 26026986 10.1007/s10899-015-9549-7

[52] Pekala RJ, Steinberg J, Kumar CK. Measurement of phenomenological experience: Phenomenology of Consciousness Inventory. Perceptual and Motor Skills. 1986; 63:983–989. doi: 10.2466/pms.1986.63.2.983

[53] Erblich J, Montgomery GH, Bovbjerg DH. Script-guided imagery of social drinking induces both alcohol and cigarette craving in a sample of nicotine-dependent smokers. Addictive Behaviors. 2009; 34(2):164–170. doi: 10.1016/j.addbeh.2008.10.007 18977604 10.1016/j.addbeh.2008.10.007 PMC2615382

[54] Reuman L, Jacoby RJ, Fabricant LE, Herring B, Abramowitz JS. Uncertainty as an anxiety cue at high and low levels of threat. Journal of Behavior Therapy and Experimental Psychiatry. 2015; 47:111–119. doi: 10.1016/j.jbtep.2014.12.002 25562749 10.1016/j.jbtep.2014.12.002

[55] Dadds M, Hawes D, Schaefer B, Vaka K. Individual differences in imagery and reports of aversions. Memory. 2004; 12(4):462–466. doi: 10.1080/09658210444000070 15487541 10.1080/09658210444000070

[56] Heiniger LE, Clark GI, Egan SJ. Perceptions of Socratic and non-Socratic presentation of information in cognitive behaviour therapy. Journal of Behavior Therapy and Experimental Psychiatry. 2018; 58:106–113. doi: 10.1016/j.jbtep.2017.09.004 29055854 10.1016/j.jbtep.2017.09.004

[57] Wright CJ, Clark GI, Rock AJ, Coventry WJ. Intolerance of uncertainty mediates the relationship between attachment and worry. Personality and Individual Differences. 2017; 112:97–102. doi: 10.1016/j.paid.2017.02.039

